# Plant-Pollinator and Plant-Florivore Interactions in Two Savanna Species of Malpighiaceae

**DOI:** 10.3390/plants14162519

**Published:** 2025-08-13

**Authors:** Ludimila Juliele Carvalho-Leite, Helena Maura Torezan-Silingardi

**Affiliations:** 1 Programa de Pós-Graduação em Ecologia e Conservação da Biodiversidade, Instituto de Biologia, Universidade Federal de Uberlândia, Uberlândia 38405-315, Minas Gerais, Brazil; 2 Programa de Pós-Graduação em Entomologia, Faculdade de Filosofia, Ciências e Letras de Ribeirão Preto, Universidade de São Paulo, São Paulo 14040-901, Brazil

**Keywords:** bee, Brazil, Cerrado, floral herbivory, grouping, isolation, Malpighiaceae

## Abstract

Plant density influences interspecific interactions such as pollination and herbivory. In denser populations, pollinators find flowers more easily, increasing reproductive success and population growth. However, the same floral attractiveness also favors floral herbivory, a relationship described by Janzen and Connell as negative density dependence, considered an important mechanism for maintaining tropical diversity. This study analyzed the reproduction of *Peixotoa tomentosa* A. Juss. (Malpighiaceae) and *Byrsonima intermedia* A. Juss. (Malpighiaceae), considering population density and its influence on pollinator and herbivore attraction. The central hypothesis was that density affects fruit production. We conducted two treatments with both species: high density and low density in a preserved Brazilian savanna. We investigated fruit production, reproductive system, floral visitation rates, and the florivory rates of each species on each treatment. Our results showed that fruiting increased with density in both species. *Peixotoa tomentosa* is an agamospermous species, while *B. intermedia* is self-incompatible and relies exclusively on pollinators. Bees visited only *B. intermedia*, and the high-density treatment received more visits. Herbivores attacked more isolated *P. tomentosa* flowers. We concluded that density influences both pollination and herbivory, affecting plant reproduction, with effects mediated by the plant’s attractiveness in denser populations and by the size and quantity of flowers in single individuals.

## 1. Introduction

In the early 1970s, two independent researchers published their studies on biodiversity, yielding similar findings [1,2]. Soon, their studies began to be presented as the Janzen-Connell Hypothesis for the maintenance of tropical biodiversity [3], which is currently part of the so-called Modern Coexistence Theory [4]. This hypothesis proposes that species diversity in tropical and temperate forests [1,2,5] is maintained through population control by natural enemies, resulting in a negative correlation between the density of conspecifics located near the generating individual and the chance of the seed or young plant to survive [1,2,4,5]. Population density indicates the number of individuals of a single species within a given space, or the quantity of conspecific organisms per area. This leads to a spacing mechanism that prevents the dominance of a single species in the environment and facilitates the colonization of vacant spaces by individuals of other species [1,5]. Furthermore, it is proposed that there exists a natural mechanism for the stabilization of plant populations and their specific enemies, enabling these animals to locate and eradicate more young individuals of abundant plant species due to their greater accessibility, mainly near the mother plant [2]. Consequently, herbivores and predators do not locate uncommon species easily, so these plants may reproduce with fewer attacks, and their seeds have a greater chance of discovering suitable locations for growth.

Once the herbivory barrier in the early stages of development has been overcome, adult plants can also be affected through the consumption of diverse plant tissues, and the extent of the damage is directly correlated with the type and the amount of the consumed area [6]. For instance, the consumption of any component of buds or flowers, such as the calyx, corolla, gynoecium, or androecium, characterizes florivory [7]. This interaction has a significant impact on the reproductive success of plants and influences the selection of floral characteristics [6,8]. Florivores exert an impact on structures associated with signaling, such as petals and odoriferous glands, and on rewarding organs to pollinators as anthers and nectaries. There are several types of florivores, such as nectar robbers able to damage flowers to obtain nectar, but they do not consume the perianth; the nectar thieves that obtain nectar without damaging the flower structure, but have incompatible morphology to be considered pollinators; the pre-dispersal predators, that are insect larvae and adults that prey on ovules or seeds before dispersing or are flower parasites, and pathogens that lead to plant diseases [6]. These aforementioned damages are costly for the plant, as the flowers require significant energy to be produced [6], and this impact results in a reduction in visitation rates and consequently decreases fruit production through pollination [8]. Therefore, similar to herbivory, florivory can also be influenced by the density of plants present in a population, as relevant floral traits for pollinator attraction, such as color, odor, duration, size, arrangement, and phenology of flowers, also contribute to florivorous attraction [9,10]. This assertion is substantiated by a close correlation between herbivory and florivory rates, indicating that when one increases, the other follows suit [9].

Beyond antagonistic interactions of herbivory and florivory, mutualistic relationships have also received attention regarding density and its relationship with associated biodiversity [11]. Excellent examples are observed in the pollination process through animal vectors, which is necessary for the production of seeds for the vast majority of plants [12]. A small number of angiosperm families (18.3%) depend on wind (~16%) or water (~2%) as vectors to transport pollen grains [13], mainly in high latitudes [14]. Animal pollination or biotic pollination is estimated to happen in about 90% of flowering plant species, at least to some extent [12]. In the tropical region, the proportion of animal pollination reaches 94% [15]. In the Brazilian savanna, locally called Cerrado, it fluctuates between 80% [16] and 86% [17]. This significant dependence is attributed to the substantial abundance of allogamous species that rely on pollinators for cross-pollination and fruiting. As the seedlings develop, they can experience the effects predicted by the Janzen-Connell hypothesis.

The pollinator’s attraction is enhanced by floral morphological adaptations, including color, shape, odors, and resources such as pollen, nectar, floral oil, resins, and fragrances, among others, to signal the presence of the flower to animals [18,19]. Herbivores may use these same cues to find the plant, such as the floral volatile compounds participating in the trade-offs of attracting pollinators and florivores [20]. Such characteristics also ensure the pollinator’s fidelity and stimulate new visits [18]. In this particular instance, the pollination is perceived as a consequence of the visit, as the visitor’s primary objective is to acquire resources, mainly food resources. From this perspective, it is suggested that large populations and/or populations with plants close to each other would exhibit a multiplied floral display, thereby facilitating the discovery of flowers by pollinators and enhancing the frequency of visits [21,22]. Therefore, in pollination, it is plausible that the density dependence would be positive rather than negative, as proposed by the Janzen-Connell hypothesis. The more flowers close together, the greater the attractiveness to floral visitors. This leaves space for alternative approaches, like the Allee Effect.

The Allee Effect posits that populations with a low density of conspecific individuals may experience decreases in fertility and growth rates [23,24,25]. In plants, the Allee Effect is related to ecological and genetic factors [26]. If a plant population is too small or its individuals are too isolated, the utilization of floral attractants may prove ineffective, resulting in inefficient energy expenditure for pollen exchange between individuals [22,27]. Additionally, it may result in alterations in pollinator behavior [28,29].

In highly fragmented environments, pollinator services may be compromised by their inability to access all the isolated fragments and consequently opt to visit only a few closer ones [30]. The potential impact of this isolation may be even greater when viewed from the perspective of plant reproductive systems. Self-incompatible plants (allogamous) would experience a direct impact on their fertility rates, resulting in a reduction in their population growth due to pollen limitation [31]. On the contrary, self-compatible plants (autogamous) found in fragmented environments and with limited pollinators may not experience any significant impact on their reproductive success within a brief duration. Hence, self-compatibility may offer a viable alternative to the selective pressures experienced by plants in small or isolated populations [25,32]. This is also applicable to apomictic species, which possess the capability to produce viable seeds in the absence of pollen grains [33,34,35]. However, in the long term, self-compatible and apomictic species may suffer inbreeding depression due to the low genetic variability of the population.

The presence of mutualistic and antagonistic interactions is influenced by factors such as population density, intraspecific competition, and interspecific interactions, which are regarded as sources of biodiversity in the population [19,22,36]. These interactions also can be altered by environmental changes due to events such as frost and fire, modifying the local populations and communities [37]. Hence, there are both costs and advantages associated with being a member of a plant community comprising numerous conspecific individuals, particularly for allogamous plants. For instance, an individual may experience a greater likelihood of having their flowers discovered and visited by pollinators, resulting in a boost in plant fitness. Nonetheless, this particular individual may be subject to higher rates of herbivory. When herbivores cause harm to the petals and/or reproductive structures of flowers, it may result in impairments to fitness due to the absence of their attraction strategies, such as intact petals, or the inability to produce seeds due to damage on the anthers and/or the pistil [8].

Hence, considering (i) the positive and negative dependencies of density; (ii) the interactions of pollination and floral herbivory; and (iii) the distinct levels of pollinator dependence, our overall goal is to investigate how variations in population density impact the reproduction of two species with distinct reproductive systems. Our main hypothesis is that population density affects fruit production. The specific objectives and their hypotheses are detailed in Table 1. This study can indicate whether plant density is able to modify pollination taxes, leading to both direct and indirect effects on fruit production.

**Figure 1:**
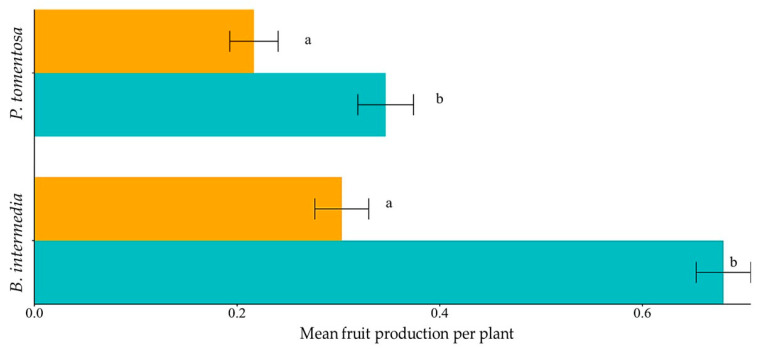
The average number of fruits produced is conditioned by plant density. The average number of fruits produced in the high-density treatment is indicated by the blue bar, and the average number of fruits produced in the low-density treatment is indicated by the orange bar. Distinct letters indicate statistical differences in each plant species between the density treatments.

**Figure 2:**
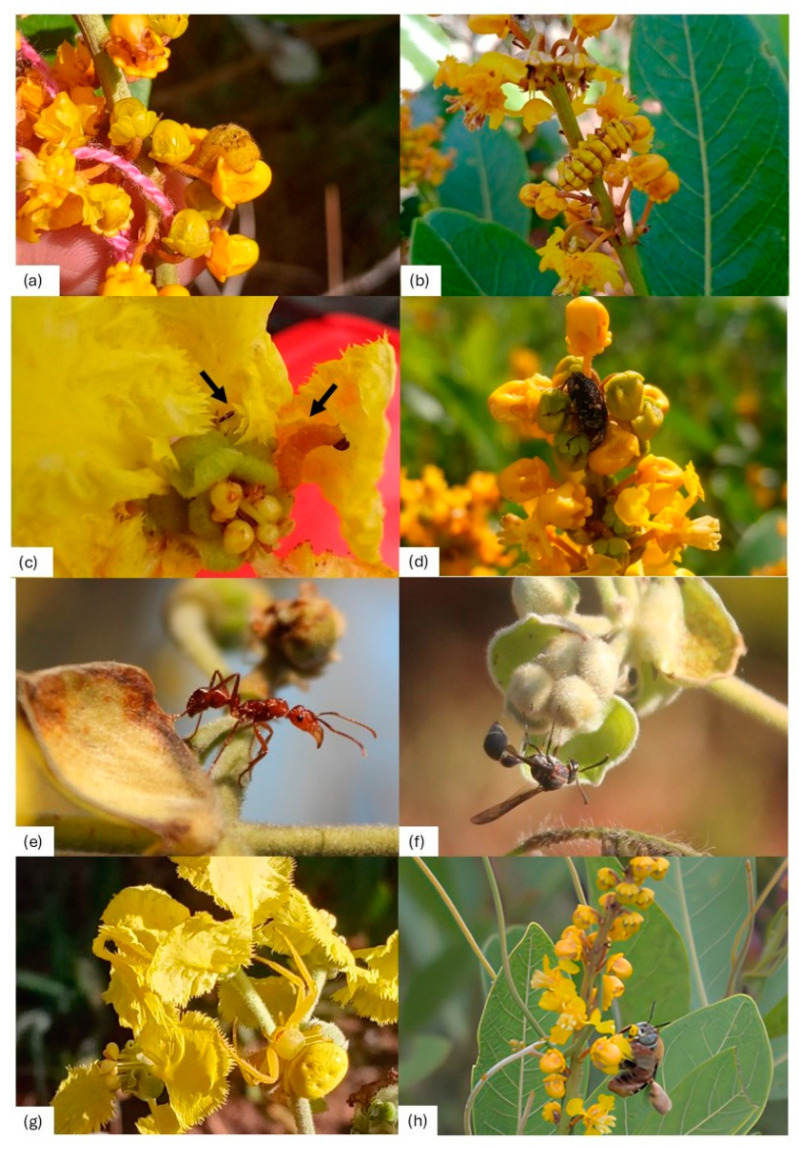
Insects visiting *P. tomentosa* and *B. intermedia* plants. Indeterminated Lepidoptera larva (**a**) and Lycaenidae-Lepidoptera larva (**b**) feeding on *B. intermedia* floral buds. Adult Thysanoptera and Lepidoptera larvae (black arrows) feeding on *P. tomentosa* petals (**c**). Curculionidae beetle in *B. intermedia* inflorescence (**d**). *Ectatomma tuberculatum* ant near flower buds and fruits of *P. tomentosa* (**e**). *Brachygastra lecheguana* wasp inspecting *P. tomentosa* floral buds (**f**). Thomisidae spider near *P. tomentosa* flowers (**g**). *Centris* sp. bee collecting oil and pollen from *B. intermedia* flowers (**h**).

**Figure 3:**
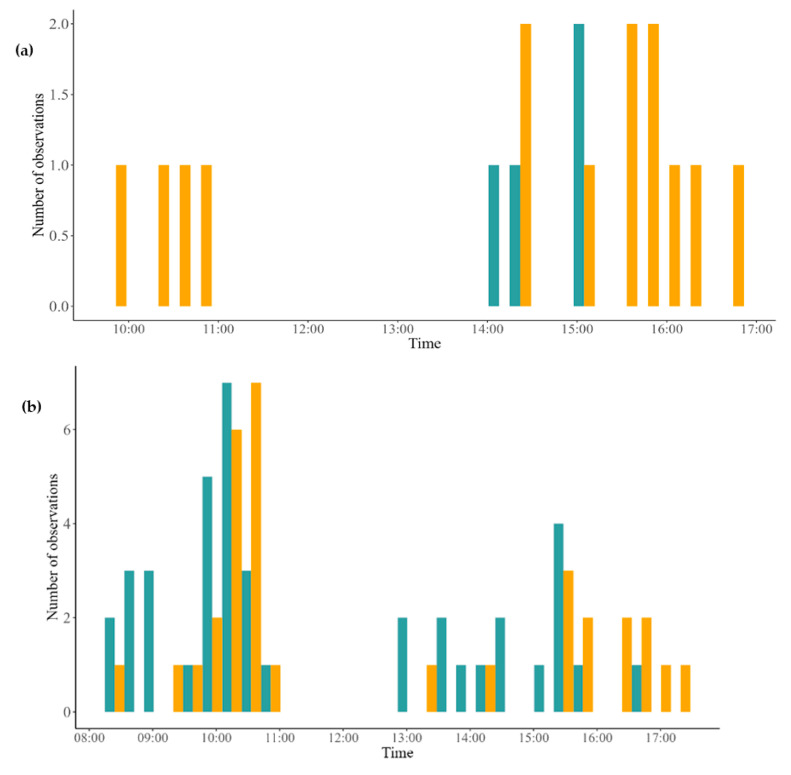
Number of visits in the flowers of *Peixotoa tomentosa* (**a**) and *Byrsonima intermedia* (**b**) conditioned by the time and by the type of treatment: high-density plants (blue) and low-density plants (orange).

**Table 2:** Fruiting of *P. tomentosa* and *B. intermedia* after different pollination treatments.

	*Peixotoa tomentosa*		*Byrsonima intermedia*	
Treatment	N Flowers/N Fruits	Fruit Set (%)	N Flowers/N Fruits	Fruit Set (%)
Agamospermy	46/16	34.78	29/1	3.44
Spontaneous Self-pollination	46/5	10.86	30/0	0
Manual Self-pollination	41/9	21.95	28/0	0
Cross-pollination	31/5	16.12	30/24	80
Control	50/5	10.0	30/17	56.6
ISI	21.95/16.12 = 1.36		0/80 = 0	
IRE	10/16.12 = 0.62		56.6/80 = 0.71	

**Table 3:** Richness and abundance of floral visiting bees and resources collected by species according to density. The acronyms indicate pollen collection by scraping (PS), pollen collection by vibration (PV), oil collection (O), and behavior capable of pollinating (*).

Floral Visitor Tribe, Species	*Peixotoa tomentosa*		*Byrsonima intermedia*		Resource Collected
	High Density	Low Density	High Density	Low Density	
**Apini**					
*Apis mellifera*	-	1	-	-	PS
**Centridini**					
*Centris flavifrons*	-	-	1	-	O *
*Centris* sp.	-	-	1	1	PV */O *
*Epicharis bicolor*	-	-	10	4	PV */O *
*Epicharis* sp.	-	-	13	3	PV */O *
**Exomalopsini**					
*Exomalopsis analis*	-	-	4	-	O *
**Halictini**					
*Agapostemon* sp.	-	-	2	3	PS
*Dialictus* sp.	-	-	-	1	PS
**Meliponini**					
*Paratrigona lineata*	1	1	5	12	PS
*Plebeia saiqui*	-	-	3	-	O
*Tetragona clavipes*	-	-	1	-	O
*Tetragonisca angustula*	2	3	3	1	PS/O
*Trigona spinipes*	1	4	2	5	PS/O
*Trigonisca* sp.	-	-	-	1	PS
Total Bees	4	9	46	31	

## 2. Results

### 2.1. Fruiting as a Function of the Reproductive System and Density

*Peixotoa tomentosa* A. Juss. (Malpighiaceae) flowered from late April to mid-August 2022, while *B. intermedia* A. Juss. (Malpighiaceae) flowered later, between late October 2022 and mid-February 2023. Controlled pollination tests showed that *P. tomentosa* is a self-compatible (autogamous) species that can form fruits and seeds even without pollen grains (agamospermy) or pollinators, with a significant difference between the number of fruits formed depending on the treatment (GLM X2 = 12.052, df = 4, *p* = 0.01697) (Table 2). Distinctly, the results of the controlled pollination treatments in *B. intermedia* showed that the species require pollen transfer between individuals to form fruits, so it was considered a self-incompatible species (allogamy), with a significant difference between the fruit formation of each group (GLM X2 = 96.114, df = 4, *p* < 2.2 × 10^−16^) (Table 2). The single fruit formed in the agamospermy treatment is probably due to accidental contamination by pollen from another individual during the emasculation process.

Fruiting was positively influenced by the increase in density in both species observed (Figure 1). *Peixotoa tomentosa* flowers produced 34% of fruits in the high-density treatment and 21% in the low-density treatment, with a significant difference between the means of the two groups (GLM X2 = 12.616, df = 1, *p* = 0.0003826). *Byrsonima intermedia*, in turn, produced 68% of the fruit in the high-density treatment and 30% in the low-density treatment, resulting in a higher statistical difference between the means of the two groups (GLM X2 = 87.291, df = 1, *p* = 2.2 × 10^−16^).

### 2.2. Floral Visitation Due to Density

Fifteen species belonging to the families Apidae, Halictidae, and Syrphidae were observed visiting the flowers of *P. tomentosa* and *B. intermedia*. There was neither a statistically significant difference in *P. tomentosa* between the number of flower-visiting species in the low-density (5 species), nor the high-density (3 species) treatments (GLM X2 = 0.50534, df = 1, *p* = 0.4772). This number also does not differ between *B. intermedia* low-density (16 species) and high-density (17 species) treatments (GLM X2 = 0.030308, df = 1, *p* = 0.8618). However, *P. tomentosa* plants received more visits (14 visits) in the low-density treatment than in the high-density treatment (4 visits) (t = −2.2646, df = 5.6701, *p* = 0.05669), while *B. intermedia* had both treatments with similar numbers of visits, with the low-density treatment (16 visits) and the high-density treatment (17 visits) without statistical differences (t = −0.22301, df = 17.719, *p*-value = 0.8261).

*Peixotoa tomentosa* was visited exclusively by pollen and oil robbers, while *B. intermedia* visitors were both pollinators and robber species (Table 3). The behavior able to transfer pollen observed in flowers of *B. intermedia* varied as a function of plant density, with higher species richness in the high-density treatment for both Centridini (high density: four species, low density: three species) and Exomalopsini bees (high density: one species). *Byrsonima intermedia* flowers were visited by bees as *Centris flavifrons* Fabricius (Hymenoptera: Apidae), *Centris* sp., *Epicharis bicolor* Smith (Hymenoptera: Apidae), *Epicharis* sp., and *Exomalopsis analis* Spinola (Hymenoptera: Apidae), which collected oil by scraping and pollen by vibration, being statistically more frequent in the shrubs in the high-density treatment (29 bees) than in the low-density treatment (8 bees) (GLM X2 = 4.3198, df = 1, *p* = 0.03767). *Peixotoa tomentosa* and *B. intermedia* had three species of Meliponini bees and one species of Diptera (Syrphidae) visiting their flowers in both treatments, acting as floral resource robbers. The bees *Paratrigona lineata* Lepeletier (Hymenoptera: Apidae), *Tetragonisca angustula* Latreille (Hymenoptera: Apidae), and *Trigona spinipes* Fabricius (Hymenoptera: Apidae) always collected pollen by scraping the anthers or oil by scraping the elaiophores, but the stigmas were never touched, and pollen transfer did not occur. The Syrphidae species were observed feeding on pollen directly from the anthers of both *P. tomentosa* (low-density treatment: 5 flies) and *B. intermedia* (low-density treatment: 7 flies, and high density: 3 flies).

Bees *Tetragonisca angustula* and a species of the genus *Trigonisca* were observed scraping extrafloral nectaries on leaves near the inflorescences of *P. tomentosa*. Other visitors were recorded for both species, such as ants (*Camponotus crassus* Mayr (Hymenoptera: Formicidae), *C. blandus* Smith (Hymenoptera: Formicidae), *Ectatomma tuberculatum* Olivier (Hymenoptera: Formicidae)), and wasps of the families Sphecidae, Pompilidae, and Vespidae (subfamilies Eumeninae and Polistinae), especially *Brachygastra lecheguana* Latreille (Hymenoptera: Vespidae) and *Polistes versicolor* Olivier (Hymenoptera: Eumenidae). Ants and wasps were recorded with mutualistic behaviors of induced defense through NEFs by ants in *P. tomentosa*, and predation of herbivores by wasps in both species. Thomisidae spiders were observed with predatory behavior only in *P. tomentosa*. Petals of *P. tomentosa* were eaten by lepidoptera caterpillars (Geometridae) on a plant at the low-density treatment, and by crickets (Orthoptera: Romaleidae) and beetles (Curculionidae) on individuals of the low-density treatment. Petals of *B. intermedia* were eaten by lepidoptera caterpillars (Lycaenidae) in a plant at the high-density treatment. These florivores caused damage to the reproductive structures (gynoecium and androceum) and the attractive structures (petals) of both open flowers and developing buds (Figure 2a–d). This damage was observed in parts of the petals or even in the flower completely, which was aborted by the plant later. Predators like ants (Figure 2e), wasps (Figure 2f), and spiders (Figure 2g) near flowers and buds can predate on pollinators (Figure 2g) and/or florivores.

The number of flowers visited varied by treatment in *P. tomentosa*, with more visits in the low-density treatment (18 flowers) than in the high-density treatment (3 flowers) (F = 8.301, df = 1, *p* = 0.009567). On the other hand, the number of floral visits in *B. intermedia* did not differ between the low-density (101 visits) and high-density (107 visits) groups (F = 0.0399, df = 1, *p* = 0.8439). *Peixotoa tomentosa* and *B. intermedia* were visited at similar times in the morning and afternoon (Figure 3). Individuals in the high-density treatment of *P. tomentosa* were only visited between 14:00 and 15:00 h, while individuals in the low-density treatment had flower visitors between 10:00 and 11:00 h and from 14:00 to 17:00 h. *Byrsonima intermedia*, on the other hand, showed two peaks of visitation in both treatments, with a higher frequency in the morning (10:00–11:00 h) and a lower one in the afternoon (15:00–16:00 h).

### 2.3. Investigating Florivory as a Function of Density

**Figure 4:**
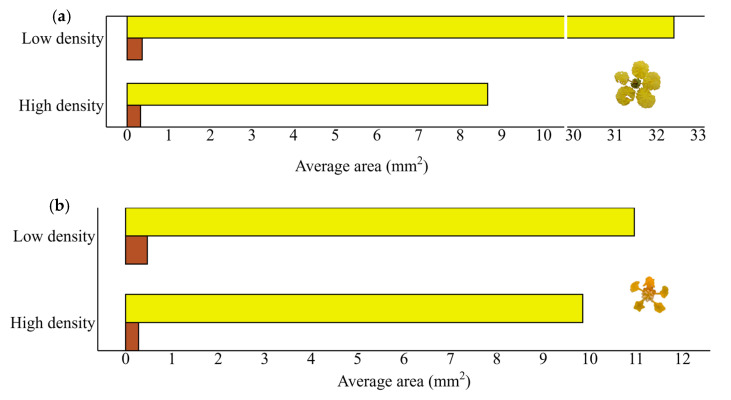
Condition of the petals. Intact area (yellow) and area consumed by florivores (brown) in *P. tomentosa* (**a**) and *B. intermedia* (**b**) according to density treatments. Each treatment featured 100 flowers.

The petals of both species were partially eaten by florivores regardless of density (Figure 4). In *P. tomentosa*, 36% of the grouped and 55% of the isolated flowers showed damage (GLM X2 = 7.3253, df = 1, *p* = 0.006799). However, the area consumed both in the high-density (0.32263 mm^2^ ± 0.3851515 mean ± standard deviation) and low-density treatments (0.36260 mm^2^ ± 0.4476348) did not differ statistically (F = 0.0659, df = 1, *p* = 0.7977). In *B. intermedia,* 71% of flowers were damaged in both treatments, and the area consumed by florivores in high-density (0.27863 mm^2^ ± 0.4797141 mean ± standard deviation) and in low-density treatments (0.46769 mm^2^ ± 0.4797141) were not statistically different (F = 1.8151, df = 1, *p* = 0.1795), similarly to *P. tomentosa*. We highlight that the flowers of *P. tomentosa* from low-density treatment plants had petals with a larger area (32.41350 mm^2^ ± 0.5012547 mean ± standard deviation) than those from high-density plants (8.659931 mm^2^ ± 0.5012547), with statistical difference between them (F = 529.81, df = 1, *p* = 2.2 × 10^−16^). However, the petal’s area of *B. intermedia* did not differ when comparing the high-density (9.851479 mm^2^ ± 0.5012547 mean ± standard deviation) and low-density (10.962989 mm^2^ ± 0.5012547) treatments (F = 2.8629, df = 1, *p* = 0.09229).

## 3. Discussion

The results confirm our first hypothesis, population density determines fruiting, but this occurs differently in autogamous/agamospermic plants and allogamous plants. The second hypothesis was confirmed as the degree of isolation of the plants influenced the visitation rates of insects in the branches, but with divergent results for *P. tomentosa* and *B. intermedia*, especially when we evaluated the visitation of pollinating bees. The third hypothesis was partially confirmed, as the relationship between population density and florivory had different results for *P. tomentosa* and *B. intermedia*.

*Peixotoa tomentosa* and *B. intermedia* showed flowering times compatible with those already reported in the literature [38,39,40]. Based on the manual pollination data, it was found that *P. tomentosa* is a self-compatible species (ISI = 1.36) that can form fruits even without pollen grains, characterizing agamospermy. This is an uncommon type of reproduction in Malpighiaceae species, just observed in three other species: *Peixotoa reticulata* Grisseb. [41,42], *Diplopterys pubipetala* (A. Juss.) W.R.Anderson & C.Davis [43,44], and *Byrsonima rotunda* Griseb [45]. The fact that cross-pollination resulted in fewer fruits than manual self-pollination may be attributed to the use of pollen from three different donors, which may have led to severe clumping of the grains inside the stigma and excessive entanglement of the pollen tubes in the stylet, resulting in competition and preventing the pollen from reaching the ovary properly [46].

Although agamospermy is rare, self-compatibility is well documented for Malpighiaceae. It is estimated that 47% of the species are self-compatible [47], with records in the literature for species of the genera *Pterandra* [48], *Malpighia* [49], *Banisteriopsis* [40,42], *Stigmaphyllon* [42], *Peixotoa* [40], *Heteropterys* [43], *Tetrapteris* [43], and *Byrsonima* [45,50,51,52], including one single study in *B. intermedia* through manual self-pollination [53]. Most literature results are similar to those presented in this study, since here *B. intermedia* is considered completely self-incompatible (ISI = 0), not bearing fruit through any of the manipulations with pollen from the same individual or with agamospermy. Both species had their natural fruiting (control) lower than the fruiting obtained through manual cross-pollination, which evidences the lack of pollinators in the area, a consequence of the recovering process after the fire. Thus, it is evident that the analysis of the reproductive efficacy index should be done with caution in species from regenerating areas, as is the case presented here, since pollination services are still below what is necessary.

As expected, the higher density of conspecific individuals had a positive effect on the fruiting rates for both *B. intermedia* and *P. tomentosa*. Evidence of reduced pollinator activities at low densities of conspecific plants, resulting in decreased fruit production, was already related [27], as increased pollination rates when conspecific plants occur in high-density fragments [11]. Our study did not observe the supply of flowers by neighboring plants of other species, but other studies [11,27] added the importance of the heterospecific neighbors of the focal plant and the scale at which the results are observed, as pollination facilitation has been observed in locally dense fragments. However, they highlight that if the results are extrapolated to the landscape level, it may cause pollination dilution. Contrary to this, a similar study with *B. coccolobifolia* Kunth (Malpighiaceae) did not find a direct relationship between the degree of isolation of individuals and fruit production [52], as well as another study with orchids [26], which did not observe a significant relationship between the size or isolation of the population and the number of fruits formed or the number of viable seeds. In this latest study, the only parameter that was affected by population size was the self-pollination rate, which decreased with the increase in the density of conspecifics. This shows that larger and denser populations tend to have more cross-pollination events, resulting in higher genetic variability and preventing inbreeding depression. In the case of self-incompatible species such as *B. intermedia*, interbreeding between related individuals in sparse populations can cause negative effects on population growth rates, consequently leading to population decline (Allee effect).

As predicted by the Janzen-Connell Hypothesis for increased herbivore attraction mediated by the greater presence of conspecific individuals [1,2], additions in fruiting are attributed to maximizing pollinator attraction via visual [54,55] and olfactory perception [20,56]. Studies show that the greater abundance of open flowers increases the display of floral attraction and culminates in a positive relationship between pollinator density and the number of flowers visited [21,54,57]. In this case, the conspecific neighborhood acts as a facilitator for pollinators to encounter flowers [11,58] and generates positive results on floral visitation rates [21,59]. The attraction mechanisms of flowers are closely related to the cognitive perception of pollinators. Floral signals are the most important mechanism for the permanence of these interactions via learning and the association between floral stimulus and resource supply [60]. In addition, the greater concentration of resources in closer plants can reduce the energy expenditure in bee foraging by up to 76% since the distance between flowers is reduced, causing them to show a preference for denser fragments [61]. *Peixotoa tomentosa* shrubs have larger flowers, but just a few open per day, while *B. intermedia* has many small flowers per day and offers lots of pollen and oil to be collected.

Our results indicated a positive relationship between floral display and fruiting for *P. tomentosa* and *B. intermedia*; however, we obtained different results regarding bee visitation in these two species. The higher density of individuals was a key factor for the frequency of visits by pollinating bees, specifically in *B. intermedia*, with a significant difference in the number of records between the high-density (29 visits) and low-density (8 visits) treatments. In this case, the facilitation of *B. intermedia* flowers to be located by pollinators is explicit, which resulted in higher fruiting rates. Comparable results indicated positive effects of conspecific flower density on visitation rates of bees of the genus *Bombus* [21,59]. However, these authors pointed out that bee visitation rates may be conditioned not only by the flower density of the focal plant species but also by the density of flowering plants that coexist in the same area. This approach adds one more factor to be considered in facilitation or competition relationships that can condition plant–pollinator interactions [11,62].

During the study period, *P. tomentosa* flowers were not visited by potential pollinating bees, but only by generalist pollen or oil-robber bees. Barônio and Torezan-Silingardi [63] recorded 10 pollinating species of *P. tomentosa* in the same area, including bees of the genera *Centris* and *Epicharis* (Centridini) and the species *Lophopedia pygmaea* Schrottky (Hymenoptera: Apidae) and *Monoeca* aff *brasiliensis* Lepeletier & Audinet-Serville (Hymenoptera: Apidae). Such bees have strong associations with Malpighiaceae species through floral oil collection [43,64,65] and are recognized for nesting in the soil or in pre-existing cavities in tree branches [66]. The absence of these bees in the study area during the flowering period of *P. tomentosa* is due to the occurrence of a large fire just a few months earlier than our observations, in September 2021, when the bee nests were consumed by fire, both in the shallow soil and in the vegetation. Later, when the first plants began to flower, the bee community was still re-establishing itself. This fact has also been demonstrated in another study conducted in the same area and period as ours, following the occurrence of fire [37]. The authors demonstrated that the richness and average number of floral visitors are significantly higher 2 years after the occurrence of fire, when the bee species had enough time to recolonize the area. In addition, the low number and diversity of herbivores, as well as the presence of spiders in *P. tomentosa* plants, may also be a consequence of the fire that hit the study area. In the case of spiders, they can still be observed visiting the extrafloral nectaries in search of resources [67], which is especially important in the environment during the post-disorder recovery.

This recovery hypothesis is reinforced by the fact that representatives of the Centridini tribe have been recorded visiting *B. intermedia* flowers, approximately 1 year after the last fire event. These bees have been observed collecting oil by scraping the elaiophores at the base of the sepals and pollen by vibration. The friction of the bees’ ventral bodies in the stigmas during the collection of oil causes the rupture of cells of the stigmatic surface, making them moist, which permits the adhesion of pollen grains transported by the bee hairs [43]. Oliveira et al. [53] considered bees of the genus *Epicharis* as effective pollinators of *B. intermedia* due to the high frequency of visits, similar to the recorded in the present study. Additionally, *Exomalopsis analis* was recorded collecting oil with behavior like that of Centridini bees in denser individuals of *B. intermedia*. Sazan et al. [68] indicated this bee species as a visitor of *B. cydoniifolia* A. Juss. (Malpighiaceae) but collecting pollen directly from the anthers. Another species of the same genus, *E. fulvofasciata* Smith (Hymenoptera: Apidae), was considered a pollinator of *B. intermedia* and *B. pachyphylla* A. Juss. (Malpighiaceae). However, it was not recorded collecting oil, but rather pollen through vibration [69]. *Exomalopsis analis* can be considered an eventual pollinator of this species due to the small size of the flowers, but it may be an effective pollinator for *P. tomentosa* since this flower is larger. In a hypothetical scenario, if individuals of *B. intermedia* were able to produce flowers soon after the reported burning event, the results of visitation rate and identity of visitors could be similar to those obtained for *P. tomentosa*. However, its reproductive success would be severely affected by the absence of pollinating bees in the area, given its self-incompatibility and dependence on pollinators as pollen vectors.

Traits used to attract pollinators, such as color, odor, size, longevity, number of flowers per individual plant, and their arrangement in inflorescences, are also important to assess attack frequency and levels of florivory in plants [6]. Flowers organized in inflorescences are more attacked by florivores because they offer a greater amount of food concentrated in time and space. In the present study, we used the Janzen-Connell hypothesis approach, often used for foliar herbivores and pathogens [70], to assess whether floral herbivory levels would also be determined by flower clustering in the Cerrado, where we obtained contrasting results. Contrary to what this hypothesis postulates, flowers in isolated individuals from *P. tomentosa* had higher florivory rates than those grouped, while in *B. intermedia,* we did not identify significant effects. The results observed in *P. tomentosa* can be justified due to the dilution of predation, or predator escape mechanisms in denser populations, as suggested by the Allee Effect [25]. The dilution process in florivory is reported as the factor that allows flowers in inflorescences to be less damaged [6]. In the present study, in addition to density not being a predictor for florivory in a species that has large and numerous inflorescences, such as *B. intermedia*, these were more damaged than the small inflorescences of *P. tomentosa*. Thus, we can consider that the concentration of resources in *B. intermedia* attracts more florivores, regardless of the density of conspecifics, as was observed for pollinators. However, its high number of flowers in the same inflorescence and same individual plant allows many flowers to escape from herbivores and bear fruit, even if others are consumed by florivores.

From this point of view, flowers in the high-density treatments should have been more attacked than those in isolated plants, which were not found. Through the data on the total area of *P. tomentosa* flowers, we suppose that flowers that are more damaged in the isolated treatment may be related to the fact that they have an expressively larger area to visually attract the insects than those grouped. According to the Plant Appearance Hypothesis [71], species (or populations within species) with larger flowers suffer a higher level of damage. However, the literature review by Boaventura et al. [6] shows that few studies indicate the total size of flowers compared to the lost area; consequently, these data need to be better explored in other systems so that this hypothesis can be corroborated or refuted.

In addition, Menge et al. [72] demonstrated that the degree of isolation of individuals in a population also affects phenotypic traits, such as plant size, considering height. In their study, the height of individuals was negatively related to population density, so plants in populations with more isolated individuals were larger than those grouped. This fact leads us to hypothesize that in our study, the isolation of individuals may have altered phenotypic traits that prioritize larger flowers as a strategy to increase the chances of being located by pollinators in the middle of vegetation, far from other conspecific plants. In addition, this phenotypic plasticity of flowers has also been related to the previous attack of antagonistic organisms, such as florivores, herbivores, and pathogens [73]. In this way, plants can change a few characteristics in the morphology, colors, volatiles, and nectar and pollen production of their flowers [74] to become more attractive to pollinators. Rusman et al. [74] show that plants infested by herbivorous caterpillars had flowers 18% larger than those without herbivores, with a 7% increase in the area of their petals. This phenotypic plasticity is adaptive and indicates a response of the organism to the selective pressures exerted by herbivores and florivores. This fact corroborates the results of visitation and flower blooming of isolated individuals of *P. tomentosa* recorded here.

Adaptation after the action of these antagonistic organisms can mean mitigating losses, since florivory affects the fitness of the plant through direct and indirect effects on the number of fruits and seeds produced [75]. Direct effects are observed in those systems where the herbivore feeds on the whole flower or in the reproductive organs, while indirect effects are linked to a decrease in the visual and olfactory attraction capacity of pollinators [76]. Such reproductive results are exemplified here by the low fruit production in *P. tomentosa* flowers in isolated individuals.

## 4. Materials and Methods

### 4.1. Area and Period of Study

**Figure 5:**
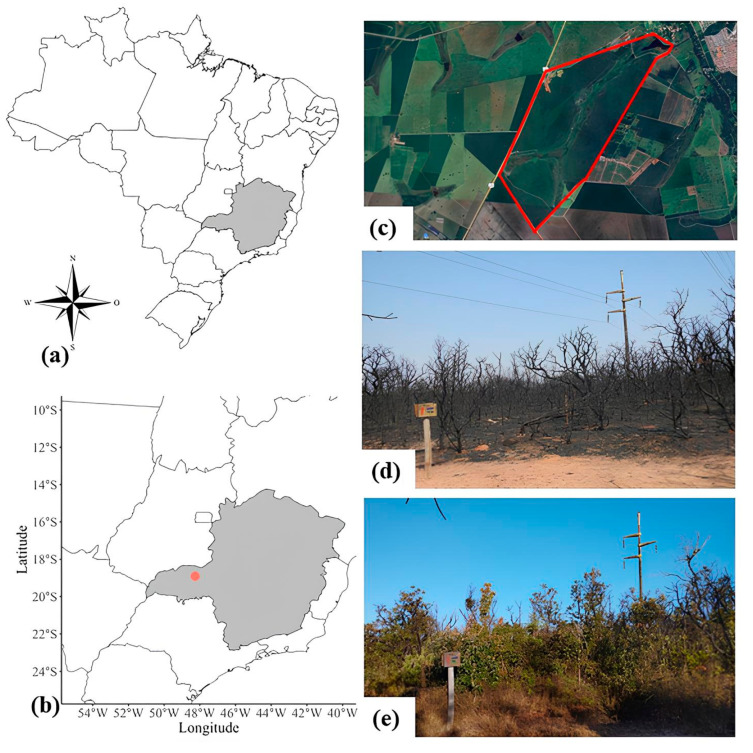
Area of study. (**a**): Minas Gerais state in southeast Brazil (gray); (**b**): Uberlândia city (red dot); (**c**): Ecological reserve of the Itororó Park Club surrounded by the red line; (**d**): Field area after the fire in September 2021; (**e**): Field area under recovery in December 2021. Sources: R Studio 4.4.2 (2024-10-31) (**a**,**b**), Google Earth (**c**), José Henrique Pezzonia (**d**,**e**).

We conducted field observations from April to December 2022 in the ecological reserve of the Itororó Park Club (18°59′569″ S–48°18′351″ W), Uberlândia city, Minas Gerais state, Brazil (Figure 5a–c). The reserve has 640 ha of Brazilian Savanna, or Cerrado vegetation, composed of physiognomies such as the Vereda, Campo Limpo, Campo Sujo, Campo Cerrado, Cerrado strictu sensu, and Cerradão [77]. The region has two defined seasons, the dry season from May to September and the rainy season from October to April, classified as Aw according to the Köppen scale [78]. The average annual temperature is 22 °C, and rainfall reaches 1600 mm. In July 2021, during a harsh winter, the reserve experienced two frost events that severely affected the vegetation. Shortly afterward, in early September 2021, an intense fire occurred (Figure 5d), destroying the vegetation that was still recovering from the severe cold. Since then, the local flora and fauna have been recovering (Figure 5e).

### 4.2. Focal Species

*Peixotoa tomentosa* A. Juss. and *Byrsonima intermedia* A. Juss. belong to the Malpighiaceae family. These species are native and endemic to Brazil, and common in Cerrado [79]. In the study area, they occur in the physiognomies Cerrado sensu strictu and Campo Cerrado, but only the ones from Cerrado sensu strictu were used in the observations. Both are woody and shrubby and produce many yellow flowers that are frequently visited by bees of the tribes Centridini, Tapinotaspidiini, and Tetrapedini [63] in search of oil and pollen [38,63]. The flowers are bisexual and zygomorphic, with five petals, one of which is differentiated into a flag petal able to sustain the bee during oil collection [64]. The calyx has oil glands (elaiophores) located between the petals, eight glands in *P. tomentosa*, and ten glands in *B. intermedia* [38,41].

These plants can present antagonistic interactions with herbivores and florivores of the orders Lepidoptera, Coleoptera, Hemiptera, Diptera, Orthoptera, Thysanoptera, and Psocoptera. Endophitic herbivores, such as the larvae of some of these insects, develop inside the flower buds and consume the reproductive structures of the flower, but they can be located and attacked by wasps [80,81]. Exophitic herbivores like beetles, hemipteran, lepidoptera, and orthoptera can also consume the floral structures and rewards [38].

**Figure 6:**
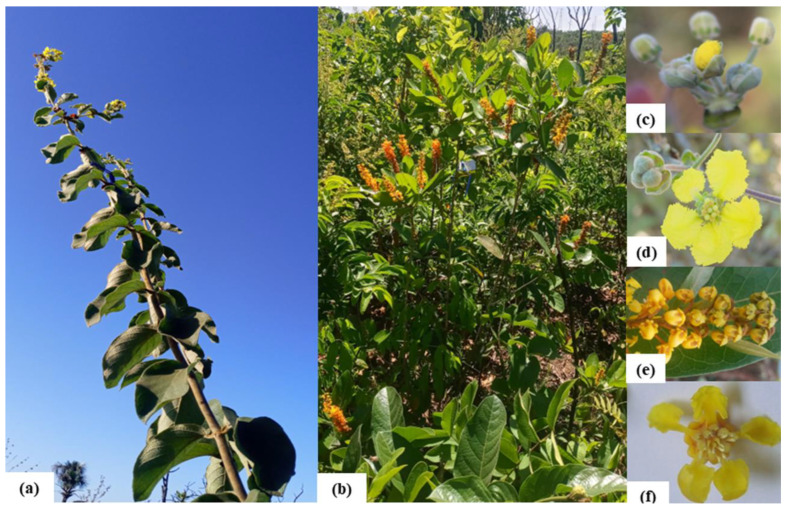
Studied species. *Peixotoa tomentosa* branch (**a**), *Byrsonima intermedia* individual plant (**b**), *P. tomentosa* inflorescence (**c**) and flower (**d**), and *B. intermedia* inflorescence (**e**) and flower (**f**).

*Peixotoa tomentosa* (Figure 6a,c,d) has a pair of extrafloral nectaries at the base of each leaf blade, near the petiole, and deciduous stipules, which are the most conspicuous feature that helps to identify the congener species [41,79]. The inflorescences are umbellate with four flowers and fringed marginal petals. The flowers have five stamens and five staminodes arranged at the same height and a three-carpellate ovary. *Peixotoa tomentosa* is an agamospermic species, able to produce fruits and seeds even if no pollen grains are present, but it produces even more fruits with pollinator services (Torezan-Silingardi, unpublished result). Each flower of *P. tomentosa* can produce a schizocarp fruit that divides spontaneously into up to three independent diaspores called samarids; each one contains only one seed within a very hard region connected to a large and light dorsal wing [82]. This dorsal wing facilitates anemochorous dispersal [83]. *Peixotoa tomentosa* can fructify even without pollen, as it is an agamouspermous species (Torezan-Silingardi, unpublished result). *Byrsonima intermedia* (Figure 6b,e,f) does not produce extrafloral nectaries and has spike-like racemose inflorescences in the terminal axil of each branch, with many flowers with wavy margin petals, 10 stamens with introrse anthers, and no staminodes [53]. *Byrsonima intermedia* is an allogamous species, highly dependent on pollinators for fruitification (Torezan-Silingardi, unpublished result). Each flower produces one fruit of the drupe type, with a single hard pyrene containing up to three seeds and a fleshy pulp that is appreciated by frugivores [82].

### 4.3. Fruiting as a Function of Reproductive System and Density

To evaluate whether the two species with different reproductive systems affect their natural fruiting in opposite ways depending on density, we first examined the reproductive system by controlled pollination tests in *P. tomentosa* (25 plants) and *B. intermedia* (15 plants). We carried out five pollination treatments, marked with colored cotton threads on the petiole: (i) agamospermy, buds in preanthesis were emasculated using fine-tipped tweezers; (ii) spontaneous self-pollination, buds were bagged; (iii) manual self-pollination, newly opened flowers manually received pollen from the flower itself; (iv) cross-pollination, newly opened flowers manually received pollen from three different individuals at least 20 m apart; (v) control, flowers were left free for natural pollination. In the first four treatments, the buds were wrapped with 100% polyester voil bags to avoid visits from pollen vectors, but the bags allowed the entry of sunlight and the gas exchanges. Manual pollination was conducted in the morning. Fruiting was recorded 1 month after the treatments.

To investigate the relationship between fruiting and population density, we conducted two treatments, each with 30 plants in an area of approximately 1.000 m^2^ for each treatment, totaling 60 individuals per species and 4.000 m^2^ in the study area. In the low-density treatment, we manipulate the natural density of individuals by selecting plants in areas where our individuals were at least 20 m away from their flowering conspecifics, and in case some plants of the same species were flowering within a radius of 20 m, all their inflorescences were cut off at an early stage of development. In the high-density treatment, we used areas with plants of the same species at a maximum distance of five meters from each other. In both treatments, 10 flower buds were marked with a cotton thread on each plant in the preanthesis phase, for a total of 300 flower buds per treatment, 600 per species, and 1200 in the entire experiment. Thirty days after the flowers opened, we counted the fruits naturally formed.

The statistical analyses were performed with the R 4.2.3 software [84]. In this and all subsequent analyses, we checked the agreement of the data with the assumptions of normality and homoscedasticity using the “DHARMA” package (version 0.4.7) [85] and the quality of the model based on the distribution of the residuals using the “performance” package (0.13.0) [86]. For all analyses, we assumed a significance level of *p* < 0.05 for the difference between the mean values [87].

To characterize the predominant reproductive system of the two species, we used a generalized linear model (GLM) with a binomial distribution, followed by a chi-square test to evaluate whether the number of fruits formed was different in each manual pollination treatment. Subsequently, the index of self-incompatibility (ISI) was calculated by dividing the percentage of fruits formed by manual self-pollination by the percentage of fruits formed by cross-pollination, with values below 0.25 indicating self-incompatible species [88]. The index of reproductive efficacy (IRE) was also calculated by dividing the percentage of cross-fertilization by the percentage of natural fertilization [89]. This index indicates the result of fruit set under the best pollination conditions. To evaluate the answer to hypothesis one, whether conspecific density affects fruiting in both species, we used another GLM with binomial distribution, since the data indicated the absence or presence of fruits (0 and 1, respectively).

### 4.4. Visitation as a Function of Density and Visitor’s Function

To evaluate whether plants near their conspecifics (high-density) were visited more than isolated plants (low-density), we conducted focal observations on 10 individuals per treatment and on plants already used to evaluate the first hypothesis in each species. Each plant was observed for 1 h in the morning and 1 hour in the afternoon, always on sunny days, with 20 h per treatment, 40 h per species, and 80 h in total. The time of arrival of each visitor in the flower, the number of flowers it visited, the presence of agonistic interactions with other visitors in the flower or plant, the part of the flower that was contacted, and the behavior of the visitor were recorded to characterize it as a pollinator or pillager of resources. Visitors were photographed, filmed, collected, and stored in plastic jars for later identification.

To compare whether there was a difference in the number of flower visitors between treatments, we used GLM with Poisson distribution followed by a type II ANOVA to assess the significance of the predictors of the model. The number of visits for each treatment was analyzed with a T-test as these data met the assumptions of normality and homoscedasticity. To assess the number of flowers visited by pollinators between treatments, we conducted an ANOVA analysis in randomized blocks that included each plant as a random factor using the “lmerTest” package (version 3.1-3) [90].

### 4.5. Florivory as a Function of Density

To assess whether florivory is dependent on the degree of isolation of the plants, we marked 10 intact and pre-anthesis flower buds on 10 plants in the low- and high-density treatments already used to test the first hypothesis, totaling 100 buds per treatment and 200 per species. The integrity of the floral structures was checked on the 2nd day after anthesis to determine whether the petals or reproductive structures had been damaged by herbivory. On this occasion, just after anthesis, the flowers were packed in plastic containers and transported to the laboratory, where they were photographed on white paper. The photos were then evaluated using ImageJ software (version 1.54d) to analyze the total area and the area lost in the case of damaged flowers; the unit of measurement used was square millimeters (mm^2^).

To evaluate whether the number of flowers damaged by florivores is influenced by conspecific density, we performed a GLM with binomial distribution followed by a type II ANOVA to assess the significance of the predictors of the model. To assess whether the mean value of damaged flower area is affected by the density of the populations in the treatments, we performed an ANOVA in randomized blocks, where we included each plant as a random factor by using the “lmerTest” package [90]. Using the same approach, we evaluated whether the total area of these flowers differed between isolated and clustered plants.

## 5. Conclusions

This study showed that density is a key factor capable of altering pollination with direct and indirect consequences on fruiting; thus, denser plants had higher fruiting than isolated ones, even considering species with distinct dependence on pollinators. Autogamous and agamospermic species, as well as allogamous species, show an increase in fruiting due to the increase in plant density. Areas recovering after a severe environmental disturbance may present insufficient pollinators, as indicated in the present study by the higher fruiting rate found in manual pollinations, evidencing that the number of pollinators in the area was still insufficient. The richness of floral visitor species was not different between more or less dense plants. However, the pollinator richness was statistically higher in *B. intermedia* (which had a later flowering in October/2022) than in *P. tomentosa*, as *P. tomentosa* produced flowers just after the fire. In *B. intermedia*, the greatest fruiting in grouped individuals is related to the facilitation of the attraction predicted by the Allee Effect. The abundance of bees was similar among plants of the same species, regardless of density. However, the species that flowered at the beginning of the recovery process (*P. tomentosa*) had a small number of visiting bees, compared to the species that flowered a few months later (*B. intermedia*), indicating the re-colonization process was in progress. Floral damage caused by herbivores was present in both species, but with high intensity and statistical difference only for the low-density *P. tomentosa* individuals, which had more flowers attacked in isolated individuals, contrary to what was proposed by the Janzen-Connell Hypothesis. It is possible that 20 h of observation in each treatment were not enough to investigate our hypothesis. We suggest that further studies investigate how the density of focal plants, as well as the presence of other flowering species in the surroundings, alter the number and richness of floral visitors, whether mutualists or antagonists. These studies may also consider plants with different pollination systems, with a distinct need for pollinating animals.

## Figures and Tables

**Table 1:** Representation of secondary hypotheses (H), specific objectives (O), predictions, statistical analysis, and results represented by figures and tables.

Secondary Hypotheses and Specific Objectives	Prediction	Statistical Analysis	Results
**H1.** *Fruiting is differently affected by population density depending on the plant’s reproductive system.* O1. Determine the type of reproductive system of two plant species. O2. Evaluate fruiting under the influence of population density.	Autogamous and agamospermous plants will present more similar fruiting rates regardless of density, unlike allogamous plants.	GLM’s, Self-Incompatibility Index and Reproductive Efficacy Index	Table 2 Figure 1
**H2.** *The visitation rate is dependent on the degree of isolation of the plants.* O3. Check the number of floral visits in populations with different plant densities. O4. Identify floral visitors as pollinators or pillagers.	Plants close to their conspecifics will be visited more than isolated plants.	GLM T-test Randomized block ANOVA	Table 3 Figure 2
**H3.** *The florivory rate is dependent on the degree of insulation of the plants.* O5. Determine the number of flowers damaged by florivores and the area consumed by florivores, considering the density of conspecific plants.	Florivores will affect grouped plants in greater quantity and intensity than isolated plants.	GLM Randomized block ANOVA	Figure 3

## Data Availability

All data is provided in the article.
